# Association between metabolic syndrome and uric acid: a systematic review and meta-analysis

**DOI:** 10.1038/s41598-022-22025-2

**Published:** 2022-11-01

**Authors:** Elena Raya-Cano, Manuel Vaquero-Abellán, Rafael Molina-Luque, Domingo De Pedro-Jiménez, Guillermo Molina-Recio, Manuel Romero-Saldaña

**Affiliations:** 1 Department of Nursing, Pharmacology and Physiotherapy, University of Córdoba, Córdoba, Spain; 2 Lifestyles, Innovation and Health (GA-16), Maimonides Biomedical Research Institute of Cordoba (IMIBIC), Avd. Menéndez Pidal N/N, 14004 Córdoba, Spain; 3 Faculty of Nursing, University of Cádiz, Cádiz, Spain

**Keywords:** Endocrine system and metabolic diseases, Predictive markers

## Abstract

This systematic review and meta-analysis aim to provide the best evidence on the association between metabolic syndrome (MetS) and uric acid (UA) by determining the size of the effect of this biomarker on MetS. The review protocol is registered with PROSPERO (CRD42021231124). The search covered the PubMed and Scopus databases. Methodological quality was assessed with the STROBE tool, overall risk of bias with RevMan (Cochrane Collaboration) and quality of evidence with Grade Pro. Initially, 1582 articles were identified. Then, after excluding duplicates and reviewing titles and abstracts, 1529 articles were excluded from applying the eligibility criteria. We included 43 papers (56 groups) comparing UA concentrations between subjects 91,845 with MetS and 259,931 controls. Subjects with MetS had a higher mean UA of 0.57 mg/dl (95% CI 0.54–0.61) (p < 0.00001). Given the heterogeneity of the included studies, the researchers decided to perform subgroups analysis. Men with MetS have a higher UA concentration mg/dl 0.53 (95% CI 0.45–0.62, p < 0.00001) and women with MetS 0.57 (95% CI 0.48–0.66, p < 0.00001) compared to subjects without MetS. Assessment of UA concentration could provide a new avenue for early diagnosis of MetS, as a new biomarker and the possibility of new therapeutic targets.

## Introduction

Metabolic syndrome (MetS) is defined as a set of metabolic abnormalities, including dysglycaemia, central obesity, dyslipidaemia (elevated triglycerides and decreased HDL-cholesterol) and hypertension. These alterations increase the risk of type 2 diabetes mellitus and cardiovascular disease^1^. The pathogenesis of Mets is not well understood but involves complex interactions between genetic background, hormones, and environmental factors such as air pollution, toxins and nutrients^2^. Previous evidence supports that insulin resistance (IR), oxidative stress and low-grade inflammation play a central role^3^.

Chronic low-grade systemic inflammation appears to be a central mechanism underlying the pathophysiology of MetS^3,4^. This inflammation is characterised by an increase in pro-inflammatory mediators and the activation of several inflammatory pathways that are significantly associated with cardiovascular events^5^. In addition, the increased concentration of pro-inflammatory substances is primarily related to obesity, especially central obesity, resulting in altered endocrine function of visceral adipose tissue^6^.

Due to the increasing prevalence of obesity, the prevalence of MetS has grown worldwide, and it is expected to continue increasing in the coming years^7^. In this respect, the adult population with MetS is estimated between 20 and 30% in most countries^8^.Due to the complexity of MetS, with diverse influences and implications for other diseases, it is not easy to make a clear-cut distinction of the diagnostic ability of the various biomarker groups. Moreover, the subdivision has limitations: the complexity of the syndrome, interactions of various biochemical pathways and the overlap of markers^9^.

Nevertheless, some studies have shown an association between MetS and the following variables indicative of inflammatory processes: uric acid (UA), C-reactive protein (CRP), liver transaminases (ALT), erythrocyte sedimentation rate (ESR), leukocytes, among others^10–12^. Likewise, through magnetic resonance spectroscopy, different metabolites have been identified in urine, highlighting glucose, lipids, aromatic amino acids, salicylic acid, maltitol, trimethylamine N-oxide and p-cresol sulphate, which have been associated with the progression of MetS^13^.

UA is an enzymatic end product of purine metabolism in humans^14^. Hyperuricaemia is a metabolic disease caused by increased formation or reduced serum uric acid (SUA) excretion. Alterations in SUA homeostasis have been correlated with several diseases such as gout, MetS, cardiovascular disease, diabetes, hypertension and kidney disease^15^.

Although SUA levels are often associated with MetS^16,17^, hyperuricaemia is not included among the diagnostic criteria that have been proposed internationally for the definition of this pathology. However, the pro-oxidant action of hyperuricaemia may induce inflammation and endothelial dysfunction by decreasing the availability of nitric oxide, thus promoting the development of the pathologies discussed above^18–21^.

Given that the prevalence of MetS increases worldwide and raises the risk of morbidity and mortality, identifying biomarkers for the early detection of this pathology is of great importance^22^. Therefore, the main Aim is to provide the best evidence on the association between MetS and UA by determining the effect size of this biomarker.

## Methods

### Literature search and selection

A systematic review and meta-analysis were carried out, following the criteria established by the PRISMA statement^23^. The search covered the PubMed and Scopus databases. The search strategy was developed by combining the following Medical Subject Headings (MeSH) descriptors: "metabolic syndrome", "uric acid", using the Boolean operator AND. The review was carried out from 2015 to May 2021. In addition, hand searching the reference lists of included studies supplemented the tracking of the available literature. The systematic review was registered in PROSPERO with ID CRD42021231124.

### Eligibility criteria

We included longitudinal, cross-sectional, case–control and cohort studies, which investigated the association between MetS and UA. In addition, their results had to include the mean and standard deviation of the study parameters. Furthermore, only papers in English and Spanish and those articles collected data in subjects older than 18 years were considered. Finally, abstracts and unpublished studies comparing subjects with and without MetS were excluded.

### Data collection

Two authors (E.R.C. and M.R.S.) separately screened all articles obtained in the search to eliminate duplicates. Then, two other authors (D.P.J. and R.M.L.) independently read the title and abstract and applied the eligibility criteria to select the articles that were finally included in the review. Finally, a fifth authors (M.V.A.) acted as a judge in case of discrepancy. One researcher (E.R.C.) oversaw extracting the data, verified by a second researcher (G.M.R.). A third researcher (M.R.S.) resolved the disagreement in case of a tie.

The extracted articles were drawn up with a table with the main characteristics (author, year, country, study design, reporting guidelines, age of participants, MetS, Aims, conclusions).

The following data were extracted from each study: citation, details of the study population (including age and sex), study design, sample size, study, aims, the mean and standard deviation of UA in those subjects with and without MetS.

### Evaluation of the qualitative synthesis

Four authors (R.M.L., D.P.J., G.M.R. and E.R.C.) were responsible for the evaluation of the qualitative synthesis through a triple analysis:

1. Assessment of methodological quality. The STROBE (Strengthening the Reporting of Observational Studies in Epidemiology) statement^24^ was used for observational studies.
2. Risk of bias assessment. Researchers were using the Cochrane Collaboration^25^ tool included in the REVMAN 5.4.2. software, the risks of selection, conduct, detection, attrition, and reporting were analysed.
3. Assessment of the quality of evidence. With the help of the Grade Protool, the evidence profile table was developed, establishing the following levels^26^:
  High: high confidence in the match between the actual and estimated effect.
  Moderate: moderate confidence in the effect estimate. There is a possibility that the actual effect is far from the estimated effect.
  Low: limited confidence in the estimate of the effect. The actual effect may be far from the estimated effect.
  Very low: low confidence in the estimated effect. The actual effect is very likely to be different from the estimated effect.

### Statistical analysis (evaluation of the quantitative synthesis or meta-analysis)

For the meta-analysis, the Cochrane Review Manager software (RevMan 5.4.2) was used to perform the statistical calculations and create the forest plots and funnel plots. Due to the difference in effect size of the included studies, a meta-analysis was performed using the Mantel–Haenszel random-effects method according to the DerSimonian and Laird model. The difference between arithmetic means with a 95% confidence interval was used to measure effect size. The risk of publication bias was assessed using the funnel plot. Heterogeneity was analysed using the Chi-square test and the inconsistency index (I^2^). According to the Cochrane Collaboration tool, heterogeneity was classified as: unimportant (0–40%), moderate (30–60%), substantial (50–90%) and considerable (75–100%).

## Results

### Characteristics of the studies

**Figure 1:**
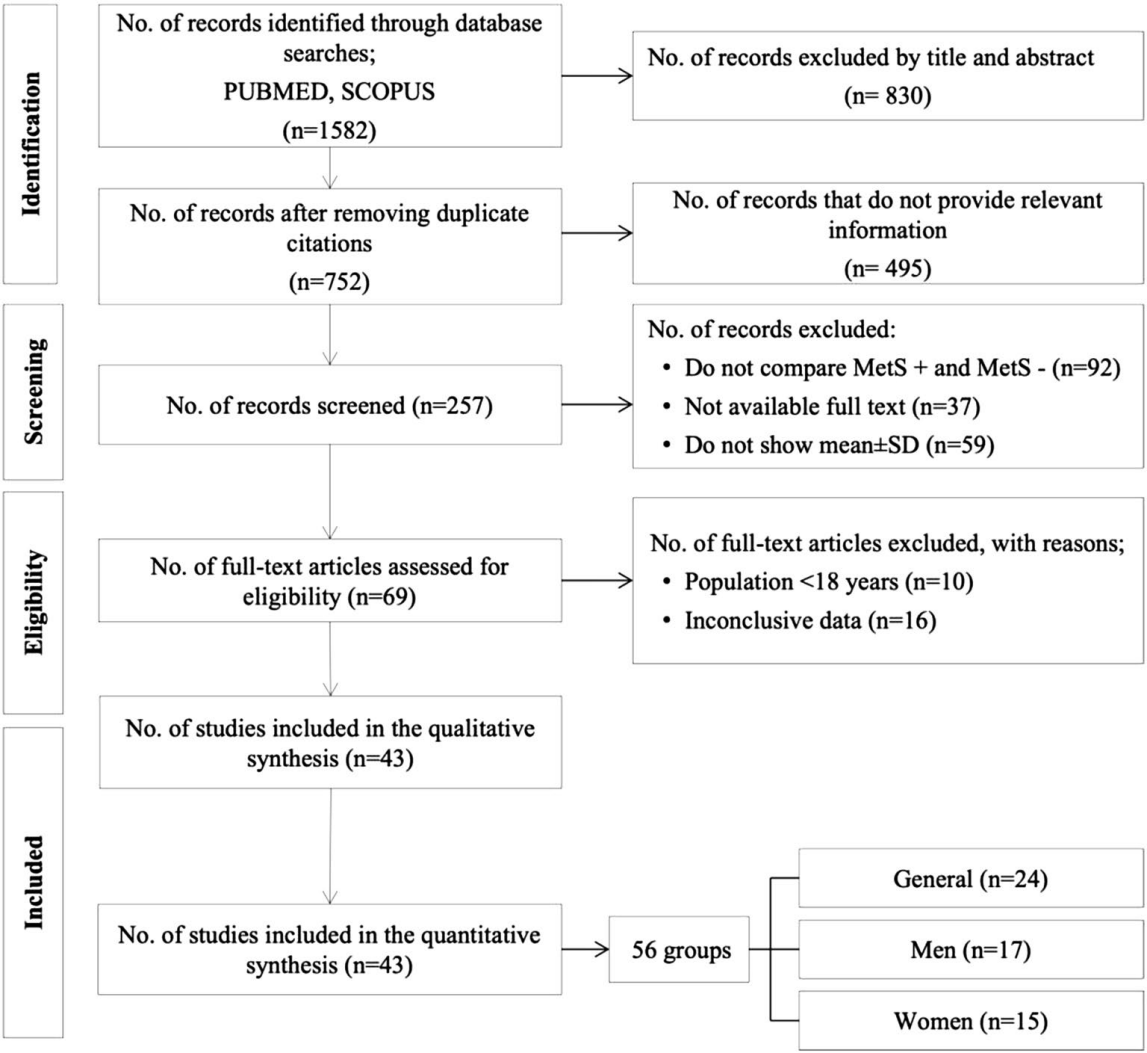
PRISMA flowchart. *MetS:* metabolic syndrome; *SD:* standard deviation.

Initially, 1582 articles were identified. Then, after excluding duplicates and reviewing titles and abstracts, 1529 articles were excluded from applying the eligibility criteria. Finally, a total of 43 articles were selected for systematic review and meta-analysis (Fig. 1).Given the large number of articles found in the search, it was divided into three subgroups: (i) articles providing UA data globally without distinction of sex (n = 24); (ii) articles with disaggregated data for men (n = 17) and (iii) women (n = 15). The detailed characteristics of the selected studies are shown in Table 1. Regarding research design, all studies were observational. Twenty-seven studies^27–53^ defined MetS according to the third report of the National Cholesterol Education Program (NCEP-Adult Treatment Panel (ATP III)^54^. Seven studies^55–61^ assessed metabolic syndrome using the International Diabetes Federation (IDF) criteria^62^. Four studies^63–66^ used the harmonised criteria^67^. Three studies^68–70^ used Chinese Medical Association criteria^71^; Sumiyoshi et al.^72^ used the Japanese criteria^73^ and, finally, Osadnik^74^ used the criteria defined in the study by Buscemi et al.^75^.

**Table 1:** Characteristics of included studies (n = 43).

Author, year, country	Study design	STROBE reporting guidelines^24^	Age of participants	No. of subjects MetS+/MetS−	MetS criteria	Aims/conclusions
Ahmadnezhad et al., 2018, Iran^55^	Cohort study	19	49.5 ± 8.1 MetS+ 47.1 ± 8.1 MetS−	2481/4727 Total 7208	IDF	Aim: association between serum prooxidant antioxidant balance (PAB), AU and hs-CRP in 7208 participants in the MASHAD study cohort Conclusion: PAB, UA and hs-CRP are independently associated with the presence of MetS
Akboga et al., 2016, Turkey^27^	Cross-sectional study	19	57.2 ± 8.7 MetS+ 55.2 ± 8.9 MetS−	114/63 Total 177	NCEP ATP III	Aim: The aim of the study was to assess the association of serum YKL-40 levels with the presence and severity of MetS Conclusion: Serum levels of YKL-40 are significantly associated with the presence of MetS
Ali et al., 2020, Bangladesh^28^	Cross-sectional study	20	39.5 ± 14.1 MetS+ 27.8 ± 10.4 MetS−	93/327 Total 420	NCEP ATP III	Aim: To assess the relationship of SUA with MetS and its components in Bangladeshi adults Conclusion: Elevated SUA is significantly associated with the prevalence of MetS and its components
Chang et al., 2019, Taiwan^29^	Longitudinal cohort study	20	≥ 30 years	409/2959 Total 3368	NCEP ATP III	Aim: to examine whether the inclusion of additional metabolic components to the current five markers can improve the discriminative validity for MetSdiagnosis Conclusion: The five current metabolic markers used for MetSdiagnosis represent the best combination with the highest discriminative validity
Chen Y et al., 2017,Taiwan^30^	Cross-sectional study	20	33.8 ± 4.8 MetS+ 30.1 ± 5.6 MetS−	2225/20,982 Total 23,207	NCEP ATP III	Aim: to investigate the relationship between UA and the presence of T2DM in the young adult population, and to determine cut-off values for UA to predict the incidence of T2DM, DM and HTN Conclusion: UA is an important predictor of the risk of developing T2DM, HT in adults, especially in the male population
Cheng et al., 2017, Italy^31^	Cross-sectional study	18	56.5 ± 16.2 Men+ 47.8 ± 18.4 Men− 56.6 ± 17.5 Women+ 44.5 ± 18.3 Women−	969/2595 Men Total 3564 1130/2676 Women Total 3806	NCEP ATP III	Aim: To explore gender differences between leukocyte telomere length (LTL) and MetS, 1999–2002 Conclusion: the more MetS components, the greater the shortening of the LTL, especially in women
Ding et al., 2018, Japan^32^	Retrospective cohort study	20	46.9 ± 9.4 MetS+ 43.5 ± 8.5 MetS−	7835/55,845 Total 63,680	NCEP ATP III	Aim: to estimate future risks of long-term health outcomes related to MetS and its components Conclusion: MetS can help identify individuals with metabolic profiles that confer substantial risk for multiple diseases, providing ancillary value in disease prediction and prevention
Fawzy et al., 2020, Saudi Arabia^33^	Cross-sectional study	20	43.1 ± 12 MetS+ 37.3 ± 16 MetS−	90/90 Total 180	NCEP ATP III	Aim: To investigate possible relationships between UA and MetS and its components in a sample of Saudi adult population Conclusion: Serum UA levels in the Saudi population may be associated with the risk of MetS and its components
He et al., 2021, China^34^	Retrospective cohort study	21	58.3 ± 7.4 Men+ 57.5 ± 7.3 Men− 57.2 ± 7.6 Women+ 53.3 ± 7.9 Women−	1339/1895 Men Total 3234 3032/3694 Women Total 6726	NCEP ATP III	Aim: association between haemoglobin levels and MetS Conclusion: haemoglobin may play an important role in the development of MetS in both men and women
Jeong et al., 2019, Korea^35^	Cross-sectional study	20	49.8 ± 0.5Men+ 43.8 ± 0.4 Men− 58.9 ± 0.6 Women+ 44.4 ± 0.4 Women−	790/1712 Men Total 2502 809/2447 Women Total 3256	NCEP ATP III	Aim: to identify optimal AU level limits for MetS prediction Conclusion: Among Korean adults, SUA levels were found to be strongly associated with the presence of MetS
Kawada et al., 2015, Japan^36^	Cross-sectional study	18	43.7 ± 7.2 MetS+ 42.4 ± 6.8 MetS−	862/4240 Total 5102	NCEP ATP III	Aim: To examine the association between MetS and biomarkers, including CRP, UA and plasma fibrinogen levels, in combination with lifestyle factors Conclusion: CRP, UA, no regular exercise and current smoking are associated with MetS
Klongthlay et al., 2020, Thailand^63^	Cross-sectional study	20	56.2 ± 10.4 MetS+ 51.7 ± 14.2 MetS−	66/136 Total 202	Harmonised criteria	Aim: to assess the prevalence of T2DM and to investigate the relationship between T2DM and risk factors Conclusion: Decreasing SUA, promoting physical activity and smoking cessation may decrease the risk of developing MetS among Thais
Lee et al., 2016, Korea^37^	Retrospective study	21	52.1 ± 8.1 Men+ 52 ± 8.5 Men− 52.6 ± 7.7 Women+ 48.8 ± 7.2 Women−	1695/5195 Men Total 6890 744/3979 Women Total 4723	NCEP-ATP III	Aim: to determine the effect of change in bilirubin concentration on the risk of incident MetS in Korean adults Conclusion: elevated bilirubin values increase the risk of MetS
Li et al., 2016, China^38^	Cross-sectional study	20	18–79 years	691/1452 Men Total 2143 1223/2207 Women Total 3430	NCEP ATP III	Aim: to assess the relationship between SUA and MetS Conclusion: normal SUA level is a contributing clinical predictor of MetS, especially in women
Liang et al., 2020, China^39^	Prospective cohort study	16	40 ± 8.9 Men+ 37 ± 9.9 Men− 45.1 ± 9.5 Women+ 36.2 ± 10 Women−	576/1949 Male Total 2525 289/1935 Women Total 2224	NCEP ATP III	Aim: to investigate the association of MetS with the incidence of thyroid nodules in Chinese adults Conclusion: nodular thyroid disease is more common in MetS cases
Liu et al., 2018, China^64^	Cross-sectional study	19	69.5 ± 7.0 MetS+ 70.0 ± 7.6 MetS−	524/920 Total 1444	Harmonised criteria	Aim: to explore the associations between liver enzymes and the risk of MetS in older populations Conclusion: elevated liver enzyme levels are positively associated with the prevalence of MetS
Martins et al., 2021, Brazil^56^	Case–control study	17	35–65 years	30/30 Total 60	IDF	Aim: to understand the pathophysiology by assessing the oxidative status associated with inflammatory processes in patients with MetS in comparison to controls Conclusion: AChE, CRP and AU markers can be used as a focus for MetS treatment
Mukhopadhyay et al., 2019, India^40^	Cross-sectional study	18	18–60 years old	113/292 Total 405	NCEP ATP III	Aim: to find out the prevalence of UA problems and their correlation with various anthropometric and metabolic parameters Conclusion: Elevated UA in subjects with MetS
Nardin et al., 2018, Italy^57^	Cross-sectional study	19	68.4 ± 10.4 MetS+ 67 ± 11.9 MetS−	2167/2563 Total 4730	IDF	Aim: to evaluate the relationship between MetS and mean platelet volume in a large cohort of patients undergoing coronary angiography Conclusion: MetS is not an independent predictor of higher mean platelet volume
Nejatinamini et al., 2015, Iran^41^	Case–control study	20	40.6 ± 6 MetS+ 37 ± 5.5 MetS−	41/60 Total 101	NCEP ATP III	Aim: to examine the association of SUA concentrations with MetS components Conclusion: people with MetS have higher levels of UA, the association of UA and MetS components supports that it could be an additional component of MetS
Ni et al., 2020, China^42^	Cross-sectional study	21	45.4 ± 11.7 MetS+ 37.9 ± 10.8 MetS−	100/3049 Total 3149	NCEP ATP III	Aim: to examine the association between SUA and the prevalence of MetS Conclusion: UA levels were associated with MetS and its components
Onat et al., 2016, Turkey^43^	Prospective cohort study	18	48 ± 12 Men+ 48.5 ± 12 Men− 49 ± 12 Women+ 45.8 ± 11.6 Women−	253/615 Men Total 868 293/541 Women Total 834	NCEP ATP III	Aim: to investigate different variables with respect to the independent predictive value of MetS risk Conclusion: elevated UA levels are a strong predictor of MetS in women
Osadnik et al., 2020, Poland^74^	Cross-sectional study	19	28 ± 4.4 MetS+ 26.8 ± 4.4 MetS−	70/390 Total 460	Buscemi et al. study criteria^75^	Aim: to evaluate the association between calcium, phosphorus and MetS in normal weight individuals Conclusion: calcium and phosphorus levels are significantly associated with MetS
Porchia et al., 2017, Mexico^65^	Cross-sectional study	21	47.2 ± 12.5 MetS+ 37.1 ± 12.8 MetS−	269/164 Total 433	Harmonised criteria	Aim: to determine the interaction of hyperinsulinaemia and hyperuricaemia on the prevalence of MetS Conclusion: UA and insulin increase the prevalence of MetS
Pugliese et al., 2021, Italy^44^	Prospective cohort study	20	62 ± 13 MetS+ 52 ± 16 MetS−	5100/4489 Total 9589	NCEP ATP III	Aim: to evaluate the prognostic role of SUA in patients with MetS Conclusion: SUA levels are associated with an increased risk of cardiovascular mortality independently of the presence of MetS.A threshold of cardiovascular SUA may improve risk stratification
Rhee et al., 2015, Korea^45^	Cross-sectional study	18	24–50 years	90/821 Total 911	NCEP ATP III	Aim: to identify the prevalence of METS and assess the association with clinical markers among male aviators Conclusion: low prevalence of MetS among aviators. Aviators with high ALT, AU, white blood cell counts should be screened for MetS
Sreckovic et al., 2020, Serbia^46^	Cross-sectional study	18	46.7 ± 15 Men+ 47.7 ± 16.7 Men−	21/15 Total 36	ATP III	Aim: to correlate the risk factors for METS and associated factors (HOMA-IR, CRP, AU, ALT, GGT) in patients with and without METS Conclusion: MetS patients had higher values of associated factors HOMA-IR, CRP, AU, ALT, GGT
Sumiyoshi et al., 2019, Japan^72^	Retrospective observational study	20	50.8 ± 9.5 MetS+ 48.8 ± 9.6 MetS−	899/7963 Men Total 8862 132/5799 Women Total 5931	Japan Diagnostic Criteria	Aim: to examine the association between the level of SUA and incident MetS in a Japanese population Conclusion: UA levels were independently associated with MetS
Tabak et al., 2017, Turkey^47^	Case–control study	17	30–65 years	130/50 Total 180	ATP III	Aim: to investigate whether there is a relationship between circulating irisin, RBP-4, PTX-3, IL-33 and adiponectin together with anthropomorphic and biochemical variables involved in the development of insulin resistance in MetS Conclusion: irisin, RBP-4, adiponectin and PTX-3 are characteristic of MetS, which is related to low-grade inflammation
Tao et al., 2020, China^48^	Case–control study	19	62.7 ± 7 MetS+ 62 ± 7.8 MetS−	455/457 Women Total 912	NCEP ATP III	Aim: to investigate the association between UA and creatine ratio and MetS in postmenopausal women Conclusion: the UA/creatinine ratio was significantly higher in patients with MetS than in controls
Tayefi et al., 2017, Iran^58^	Cross-sectional study	20	50.05 ± 7.9 MetS+ 46.74 ± 8.0 MetS−	3211/3367 Total 6578	IDF	Aim: to determine which of the IDF criteria is suitable for the Iranian population to identify patients with and without MetS Conclusion: suggest that the IDF criteria are adequate to identify individuals within the Iranian population into those with or without MetS
Vigna et al., 2017, Italy^49^	Cohort study	19	16–84 years	154/80 Men Total 234 300/291 Women Total 591	NCEP ATP III	Aim: to assess gender differences in UA, homocysteine and inflammatory biomarkers as determinants of MetS Conclusion: UA is positively related to MetS in both sexes
Wang et al. 2019, China^68^	Cohort study	21	68.9 ± 7.3 MetS+ 69.5 ± 8.3 MetS−	258/999 Total 1257	Chinese Medical Association	Aim: to assess the prevalence of MetS and its association with subclinical carotid atherosclerosis and cardiovascular morbidity and mortality in a Chinese population Conclusion: older adults with Mets have a significantly higher risk of subclinical carotid atherosclerosis, myocardial infarction, stroke and cardiovascular disease (CVD) death than those without MetS
Wang et al.,2020, China^50^	Cross-sectional study	19	68.7 ± 6.5 MetS+ 68.3 ± 6.5MetS−	2207/1791 Total 3998	NCEP ATP III	Aim: to investigate the association between SUA and ALT levels and the risk of MetS Conclusion: a combined increase in SUA and ALT is significantly more associated with MetS than an increase in SUA or ALT alone
Wang et al., 2021, China^69^	Case–control study	20	76.4 ± 6.9 MetS+ 75.3 ± 7.5 MetS−	100/102 Total 202	Chinese Medical Association	Aim: to elucidate the relationships between MetS, Apolipoprotein E (ApoE) and cognitive dysfunction in an elderly Chinese population Conclusion: MetS diagnosis and ApoE are independently associated with cognitive dysfunction
Wang, et al., 2018, China^66^	Cross-sectional study	19	69.34 ± 7.1 MetS+ 70.6 ± 6.7 MetS−	161/307 Total 468	Harmonised criteria	Aim: to investigate the relationship between UA and MetS in elderly women Conclusion: high UA is positively associated with the prevalence of MetS in elderly women
Wu et al., 2018, Taiwan^51^	Cohort study	20	35.7 ± 5.7 Men+ 32.7 ± 5.8 Men− 36.9 ± 5.9 Women+ 32.9 ± 6.4 Women−	2225/20,982 Men Total 23,207 115/3964 Women Total 4079	NCEP ATP III	Aim: to explore the prediction of aerobic exercise and resistance training in MetS and diabetes Conclusion: poor performance in aerobic and endurance exercise tests may be predictive of MetS and diabetes
Yang et al., 2021, China^70^	Case–control study	19	54.8 ± 12.5 MetS+ 45.6 ± 12.7 MetS−	538/5164 Total 5702	Chinese Society of Diabetes	Aim: to explore the association between MetS and biochemical profiles Conclusion: cystatin C levels were significantly associated with the incidence of MetS
Yen et al., 2015, Taiwan^52^	Cohort study	20	76.4 ± 6.7 MetS+ 75.8 ± 7.0 MetS−	31,307/42,240 Total 73,547	ATP III	Aim: to assess the effects of MetS and its components on mortality Conclusion: individual components of MetS are better predictors of all-cause and cause-specific mortality than MetS as a whole
Yu et al., 2015, Korea^59^	Retrospective longitudinal study	20	51.9 ± 8.2 Men+ 51.6 ± 8.3 Men− 52.9 ± 7.6 Women+ 48.6 ± 7.2 Women−	2974/5741 Male Total 8715 1241/4486 Women Total 5727	IDF	Aim: to investigate whether longitudinal effects of baseline SUA levels influence incident MetS while including body composition as a confounder in a large number of subjects Conclusion: elevated SUA levels are strong and independent predictors of MetS
Yu et al., 2018, Korea^60^	Longitudinal study	20	51.8 ± 7.9 Men+ 51.7 ± 8.4 Men− 52.4 ± 7.5 Women+ 48.6 ± 7.2 Women−	2012/5682 Men Total 7694 901/4462 Women Total 5363	IDF	Aim: to investigate the relationship between changes in SUA level and the development of MetS Conclusion: increased SUA independently protects against the development of MetS, suggesting a possible antioxidant role in the pathogenesis of incident MetS
Zhang et al., 2018, China^61^	Cross-sectional study	19	55.1 ± 9.9 Men+ 57.6 ± 9.8 Men− 57.4 ± 8.8 Women+ 54.4 ± 9.9 Women−	1390/4964 Men Total 6354 3998/6225 Women Total 10,223	IDF	Aim: to explore the association between SUA and MetS in rural Chinese adults Conclusion: positive association between SUA and prevalence of MetS in rural Chinese population
Zomorrodian et al., 2015, Iran^53^	Cross-sectional study	20	50.4 ± 7.9 MetS+ 46.8 ± 8.1 MetS−	2175/4317 Total 6492	NCEP ATP III	Aim: to explore the association between Mets and the risk of developing CKD in 6492 participants with and without Mets Conclusion: we demonstrate a significant association between some components of METS and increased prevalence of chronic CKD in the Iranian population

*STROBE* Strengthening the Reporting of Observational Studies in Epidemiology, *MetS* metabolic syndrome, *Dx* diagnosis, *IDF* International Diabetes Federation, *UA* uric acid, *hs-CRP* high-sensitivity C-reactive protein, *NCEP ATP III* National Cholesterol Education Program Adult Treatment Panel III, *SUA* serum uric acid, *DM* diabetes mellitus, *T2DM* type 2 diabetes mellitus, *HOMA-IR* Homeostatic Model Assessment of Insulin Resistance, *HT* hypertension, *ALT* alanine aminotransferase, *GGT* gamma glutamyl transferase, *CKD* chronic kidney disease.

Concerning the articles' origin, twelve (27.9%) were conducted in China^34,38,39,42,48,50,61,64,66,68–70^. In total, the 43 selected papers compared UA concentrations between 91,845 subjects with MetS and 259931controls. The age of study participants ranged from 18 to 90 years.

### Methodological quality assessment

All papers scored 16 points or more out of the 22 items included (highest tercile). No article was excluded for insufficient methodological quality. Table 1 shows a column with the score for each of the reports.

### Bias risk analysis

**Figure 2:**
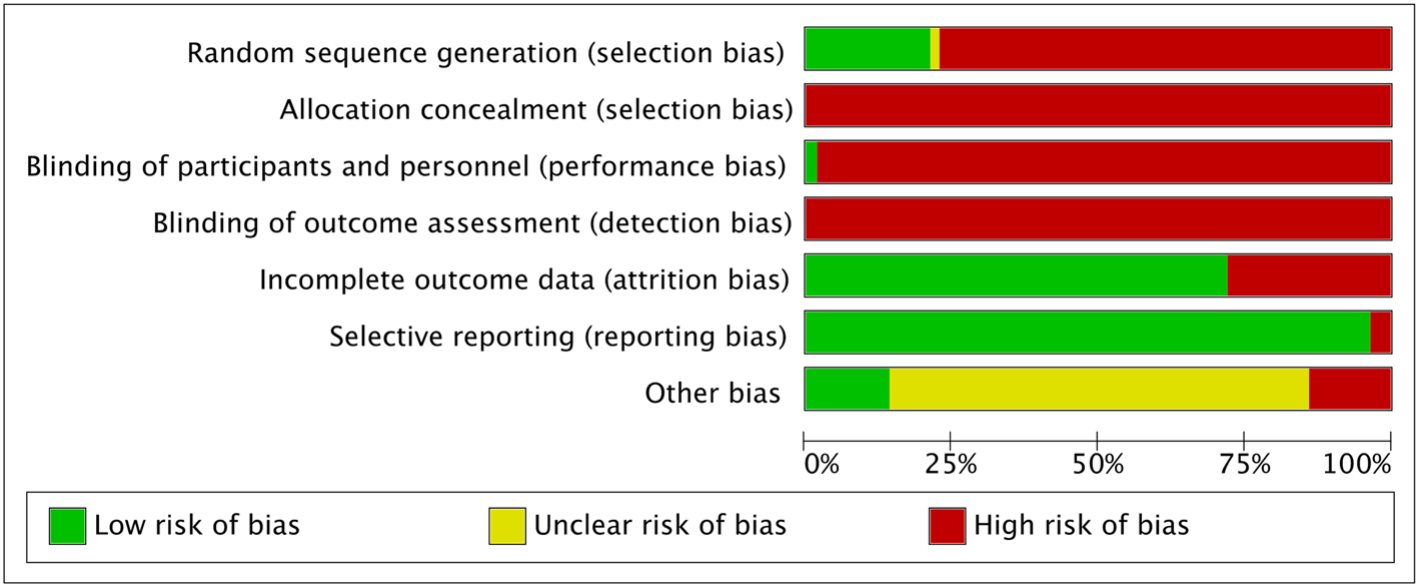
Overall risk of bias of the studies.

**Figure 3:**
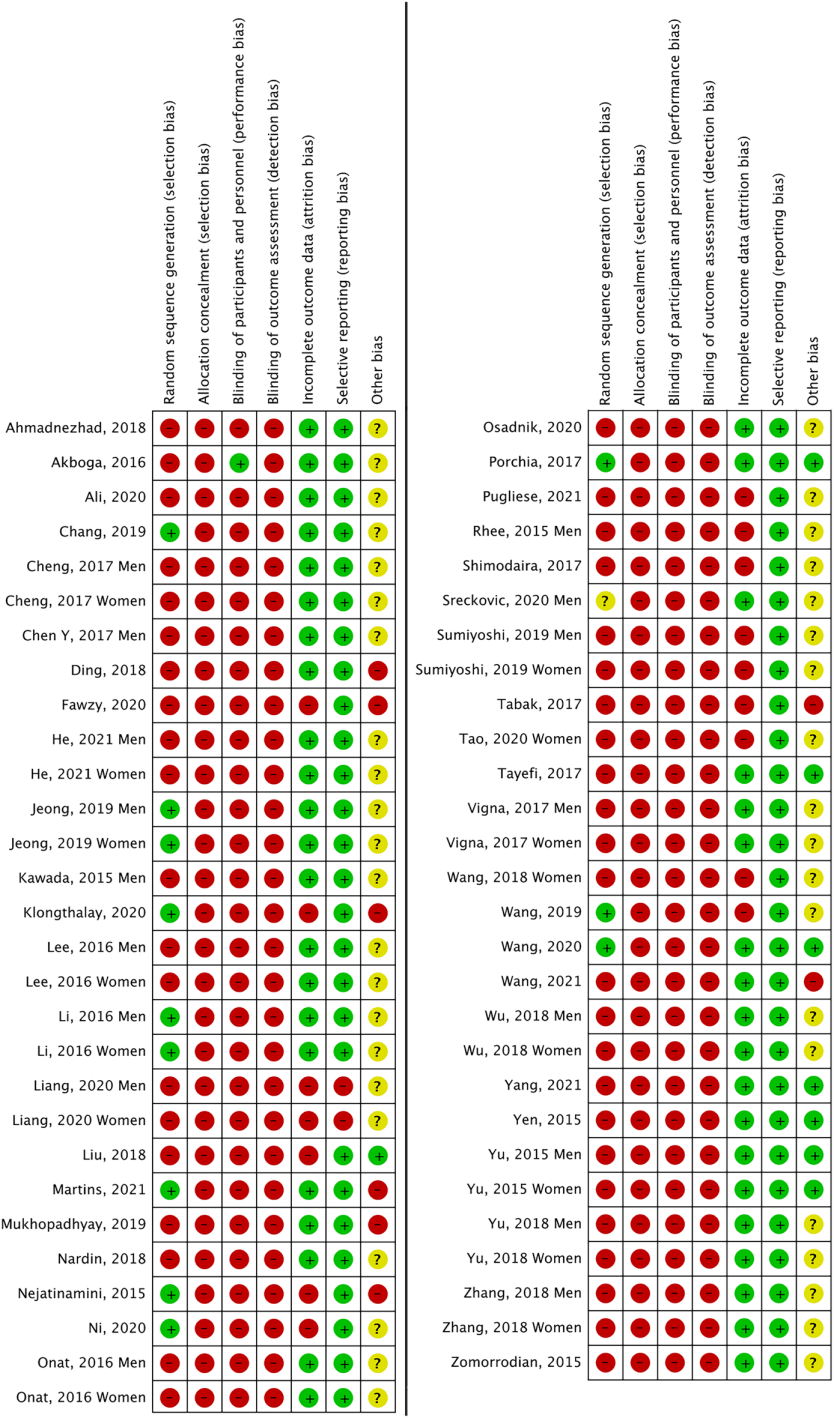
Summary of risk of bias by study.

Overall (Fig. 2), the main biases were: random sequential generation, allocation and participant and staff concealment, and blinding of outcome assessment, affecting 72% of the reports. Figure 3 represents the individual assessment of the included studies.

### Quantitative analysis. Meta-analysis

#### Meta-analysis 1

**Figure 4:**
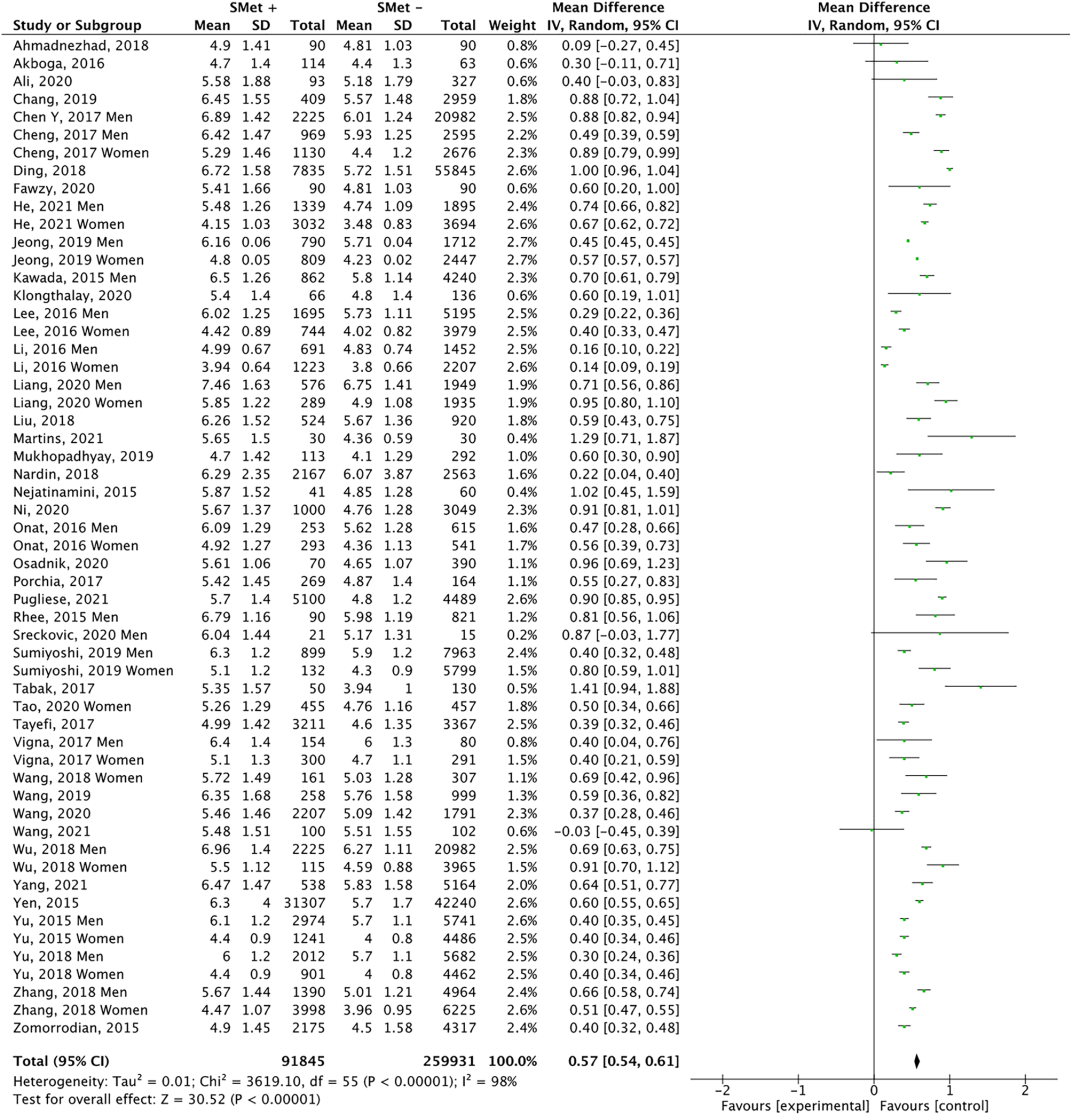
Results and summary statistics of studies analysing uric acid levels in the total population with and without metabolic syndrome (MetS) (meta-analysis 1).

**Figure 5:**
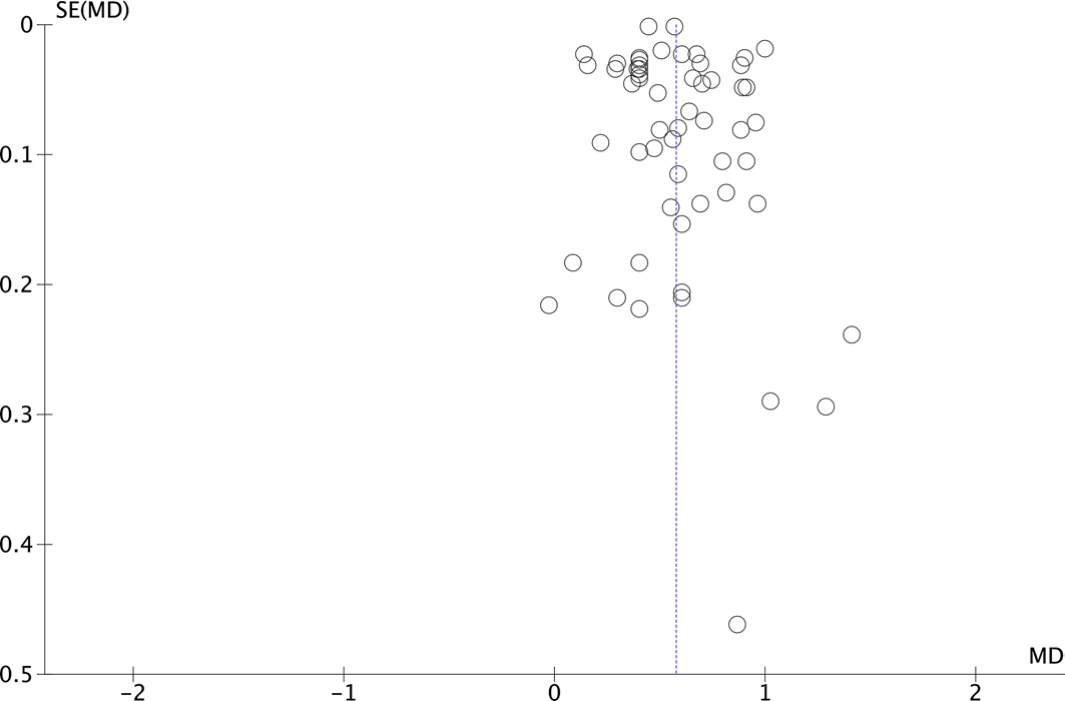
Funnel plot (meta-analysis 1).

This analysis comprises 43 papers, including men and women, together or separately, resulting in 56 groups (Fig. 4). Subjects with MetS had a mean UA 8.2% higher than those without this syndrome (5.89 mg/dl vs. 5.44 mg/dl; p < 0.00001). The funnel plot (Fig. 5) shows a low risk of publication bias. The sensitivity analysis performed to assess the pooled estimate's stability concerning each meta-analysis study did not show that any study significantly affected the heterogeneity of the meta-analysis; therefore, none was excluded. Given the heterogeneity of the included studies, it was decided to perform subgroup analysis.

#### Meta-analysis 2

**Figure 6:**
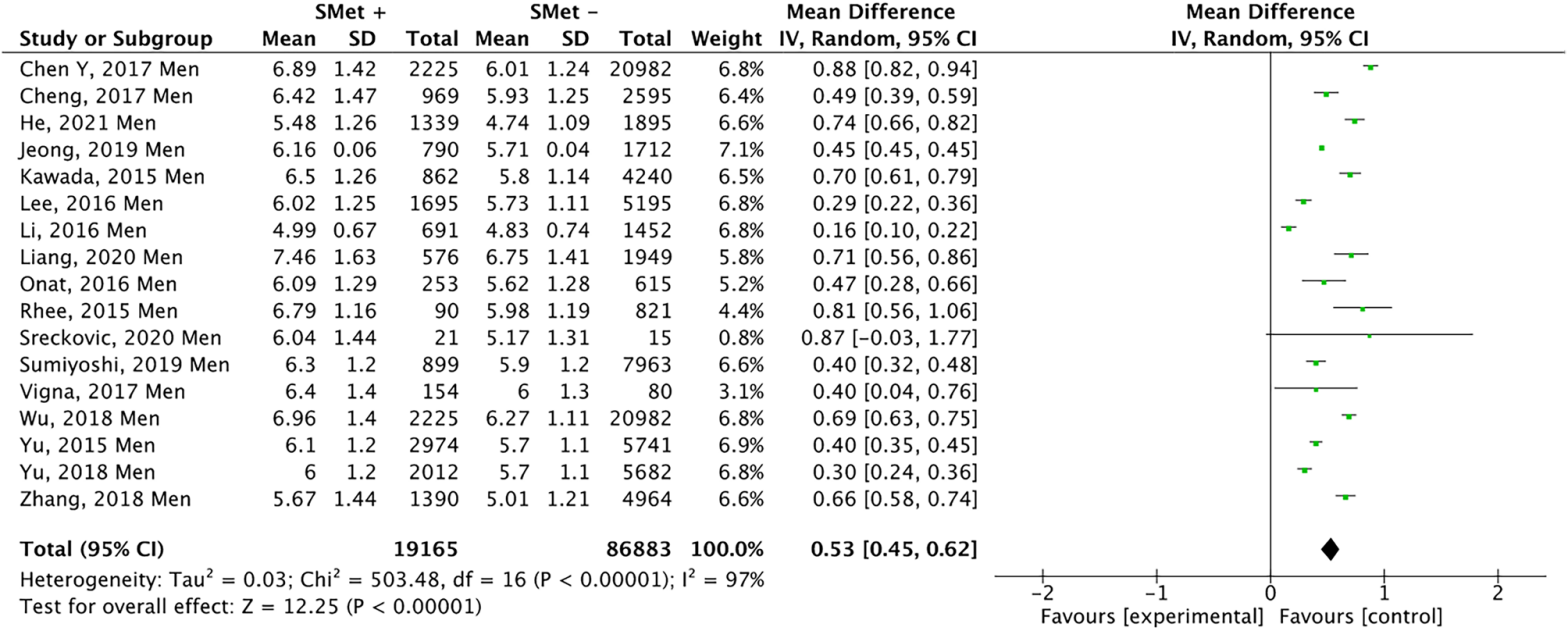
Results and summary statistics of studies analysing uric acid levels in men with and without metabolic syndrome (MetS) (meta-analysis 2).

**Figure 7:**
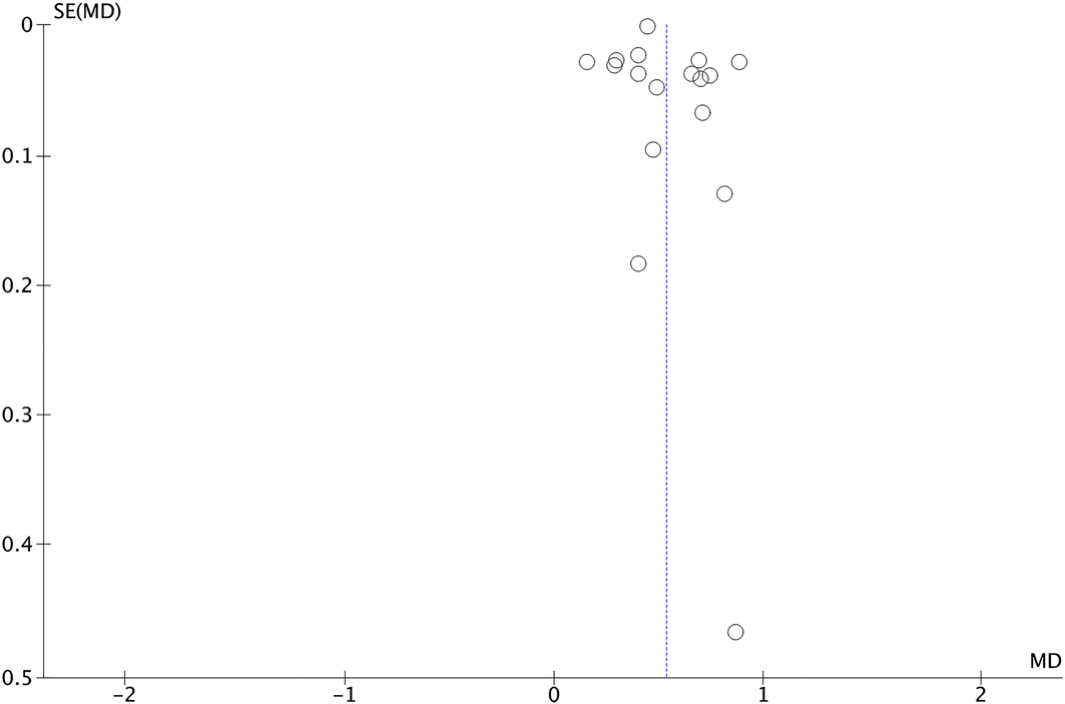
Funnet plot (meta-analysis 2).

Figure 6, which includes 17 studies, represents the results obtained when analysing the presence of UA in men with and without MetS. In this case, men with MetS showed a higher mean UA, (0.53 mg/dl; 95% CI 0.45 − 0.62; p < 0.00001; I^2^ = 97%). Figure 7 shows that there is a low risk of publication bias.

#### Meta-analysis 3

**Figure 8:**
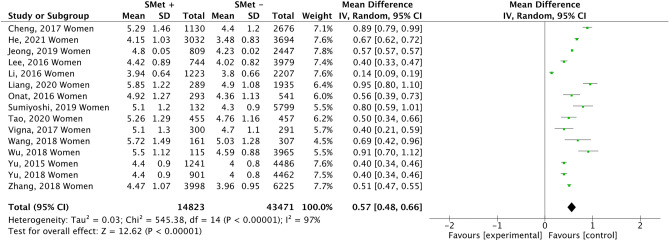
Results and summary statistics of studies analysing uric acid levels in women with and without metabolic syndrome (MetS) (meta-analysis 3).

**Figure 9:**
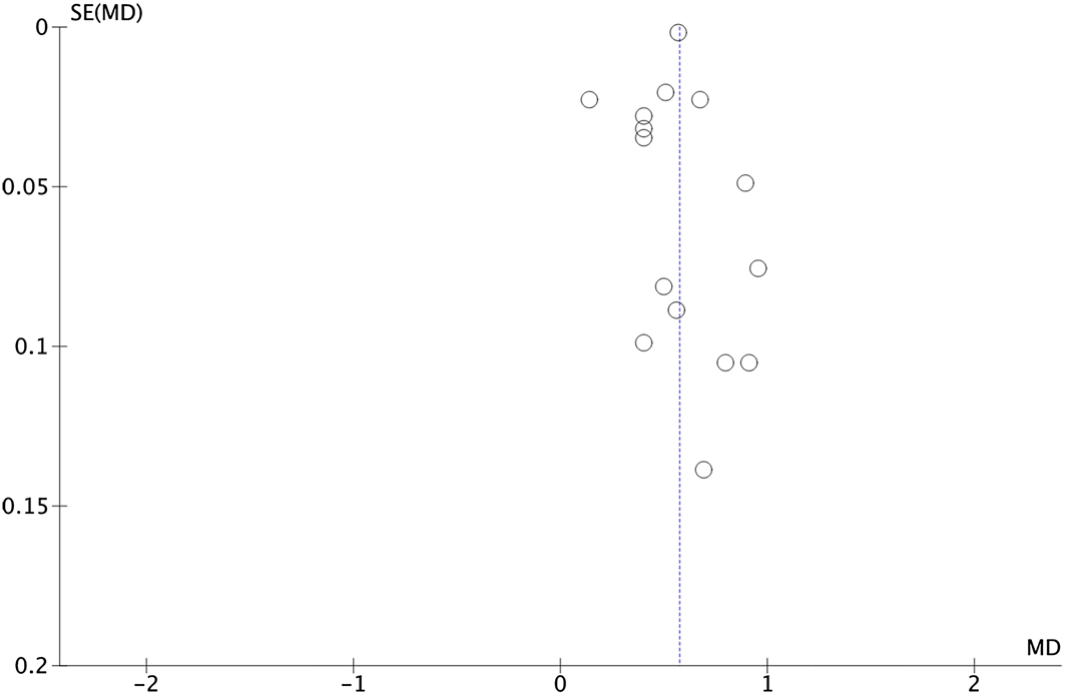
Funnet plot (meta-analysis 3).

Figure 8 compiles the results of 15 studies that examined the association between UA in women and the presence of MetS. The results show that UA level was associated with the diagnosis of METS (0.57 mg/dl; 95% CI 0.48–0.66; p < 0.00001; I^2^ = 97%). This meta-analysis also observed a low risk of publication bias (Fig. 9).

### Quality of evidence

Table 2 shows the evidence profile of the three meta-analyses, providing specific information regarding the overall certainty of the evidence of the studies included in the comparison, the magnitude of the studies examined and the sum of the data available for the outcomes assessed.

**Table 2:** Evidence profile with GRADE pro for the three meta-analyses.

Certainty assessment							No. of subjects		Size of the effect	Quality of evidence
N of studies	Study design	Risk of bias	Inconsistency	Indirect evidence	Imprecision	Other considerations	MetS+	MetS−	Difference of averages (95% CI)	
**Meta-analysis 1**										
n = 56	Observational studies		Very serious	It is not serious	It is not serious	Dose–response gradient	91,845	259,931	0,57 (0.54–0.61)	⨁◯◯◯ Very low
**Meta-analysis 2**										
n = 17	Observational studies		Very serious	It is not serious	It is not serious	Dose–response gradient	19,165	86,883	0.53 (0.45–0.62)	⨁◯◯◯ Very low
**Meta-analysis 3**										
n = 15	Observational studies		Very serious	It is not serious	It is not serious	Dose–response gradient	14,823	43,471	0.57 (0.48–0.66)	⨁◯◯◯ Very low

*MetS* metabolic syndrome, *CI* confidence interval.

## Discussion

A systematic review and meta-analysis were conducted to analyse the most recent evidence on the relationship between MetS and UA. Forty-three studies were selected, the effect size and the limitations that have conditioned the results of the different studies were quantified.

Of the included papers, 26 directly associated UA with MetS^28–30,33,35,36,38,40–46,48–50,56,57,59–61,63,65,66,72^, and 17 reports collected data indirectly^27,31,32,34,37,39,47,51–53,57,58,64,68–70,74^, i.e. they study parameters related to MetS and collect data associated with UA. These studies had limitations, but overall, all demonstrated a sufficient degree of methodological reliability and quality in terms of the association of UA and MetS.

This meta-analysis provides evidence of a relationship between UA level and MetS. The concentration of UA in subjects with MetS was significantly higher than in the control group. The meta-analysis is notable for its large sample size, with 91,845 subjects in the MetS group and 259,931 in the control group. Given the heterogeneity of the included studies, it was decided to perform subgroup analysis. The results obtained show that men with MetS have a higher UA concentration than those without MetS (mean difference (MD): mg/dl 0.53; 95% CI 0.45–0.62; p < 0.00001). This was also observed in women (MD 0.57 mg/dl; 95% CI 0.48–0.66, p < 0.00001).

Changes in the UA concentrations in human fluids can reflect the metabolic state, immunity, and other human body functions. If the concentration of UA in the blood exceeds normal, the human body fluid becomes acidic, which affects the normal function of human cells, leading to long-term metabolic disease^76^. UA correlates with obesity, diabetes mellitus^76^, hypertension^77^, cardiovascular disease^78^ and chronic kidney disease^79^, where UA acts as an oxidant, inducing oxidative stress and endothelial dysfunction^80^.

Previous studies have reported significant associations between hyperuricaemia and individual elements of the metabolic syndrome^81,82^. The study by Norvik et al.^83^ showed that elevated UA levels are associated with components of the MetS, such as hypertriglyceridaemia, insulin resistance, elevated blood pressure and low high-density lipoprotein cholesterol. Xu et al.^84^ concluded that the relationship between SUA and elevated body mass index, hypertension and hyperglycaemia varies by sex. Reducing SUA levels by adopting a healthier lifestyle may be a valuable strategy to reduce the burden of MetS^84^.

Overall, the results have shown that people with MetS have 8.2% more UA, so reducing UA could positively impact the development of this syndrome. The results found by several authors^85–87^ support this. Yuan et al.^85^, in a meta-analysis based on prospective studies of various populations, suggest that for every 1 mg/dl increase in SUA level, the risk of MetS increases by 30% with a linear dose–response relationship. Liu et al.^86^ observed a consistent and linear causality of increased UA on the incidence of MetS, concluding that SUA could be an individualised predictor in detecting systemic/hepatic metabolic abnormalities. It is estimated that people with high UA are 1.6 times more likely to develop MetS^87^. Therefore, reducing SUA levels could be a potential treatment to prevent comprehensive metabolic disorders.

At the methodological level, the assessment of risks of bias in studies is a major issue in this type of research, in line with PRISMA recommendations. Studies with similar methodologies but with discrepancies in quality may have biased results. Among all the papers included in this review, only ten studies^29,35,38,41,42,50,56,63,65,68^ had performed this step correctly. The quality of the evidence obtained is "very low" since observational studies have been analysed where there is a high risk of bias and, in addition, they present a very high inconsistency (heterogeneity).

One of the main strengths of this review is the comprehensive search that covered a wide geographical area. In addition, a large sample size of subjects with and without MetS was included, which strengthened the study's statistical power.

The interpretation of the findings in this systematic review and meta-analysis must be made considering some limitations. First, most of the studies are from China, making it difficult to generalise the results to other countries. Author bias should also be a limitation since the same research team wrote several studies. Finally, it should be noted that there is still a lack of uniformly accepted diagnostic criteria for the diagnosis of MetS.

## Conclusions

Current diagnostic criteria for MetS vary, although there is a consensus on the main components of the syndrome. None of these criteria includes UA levels in the definition of MetS.

The results have shown that UA levels are associated with the presence of MetS. In particular, subjects with MetS have been found to have higher plasma UA. The assessment of UA concentration could provide a new avenue for early diagnosis, identifying new biomarkers, and discovering new therapeutic targets.

A detailed understanding of the components of MetS is essential for the development of effective prevention strategies and appropriate intervention tools, which could curb its increasing prevalence and limit its comorbidity.

However, well-designed, high-quality randomised controlled trials are needed to confirm these findings.

## Supplementary Information

Supplementary Information.

## Data Availability

All data generated or analysed during this study are included in this published article [and its supplementary information files].
